# Unveiling ferroptosis: a new frontier in skin disease research

**DOI:** 10.3389/fimmu.2024.1485523

**Published:** 2024-10-04

**Authors:** Ke Wang, Yumeng Lin, Dan Zhou, Peipei Li, Xiaoying Zhao, Zhongyu Han, Haoran Chen

**Affiliations:** ^1^ Deyang Hospital Affiliated Hospital of Chengdu University of Traditional Chinese Medicine, Deyang, China; ^2^ Health Management Center, Naniing Tongren Hospital, School of Medicine, Southeast University, Nanjing, China; ^3^ School of Smart Health Care (School of Health & Medical), Zhejiang Dongfang Polytechnic, Zhejiang, China; ^4^ Department of Obstetrics and Gynecology, People’s Hospital of Ningxia Hui Autonomous Region, Yinchuan, Ningxia, China; ^5^ Science Education Department, Chengdu Xinhua Hospital Affiliated to North Sichuan Medical College, Chengdu, China; ^6^ Department of Gerontology, The Affiliated Traditional Chinese Medicine Hospital, Southwest Medical University, Luzhou, China

**Keywords:** ferroptosis, iron metabolism, skin diseases, melanoma, psoriasis

## Abstract

Ferroptosis, a form of regulated cell death distinct from apoptosis, necrosis, and autophagy, is increasingly recognized for its role in skin disease pathology. Characterized by iron accumulation and lipid peroxidation, ferroptosis has been implicated in the progression of various skin conditions, including psoriasis, photosensitive dermatitis, and melanoma. This review provides an in-depth analysis of the molecular mechanisms underlying ferroptosis and compares its cellular effects with other forms of cell death in the context of skin health and disease. We systematically examine the role of ferroptosis in five specific skin diseases, including ichthyosis, psoriasis, polymorphous light eruption (PMLE), vitiligo, and melanoma, detailing its influence on disease pathogenesis and progression. Moreover, we explore the current clinical landscape of ferroptosis-targeted therapies, discussing their potential in managing and treating skin diseases. Our aim is to shed light on the therapeutic potential of modulating ferroptosis in skin disease research and practice.

## 1 Introduction

In the field of pathology, skin diseases encompass a range of cell death mechanisms, including apoptosis, necrosis, and autophagy, among others (1, 2). Among these forms of regulated cell death, ferroptosis emerges as a distinctive and noteworthy type, distinguished by its unique cellular morphology and regulatory mechanisms.

**Figure 1:**
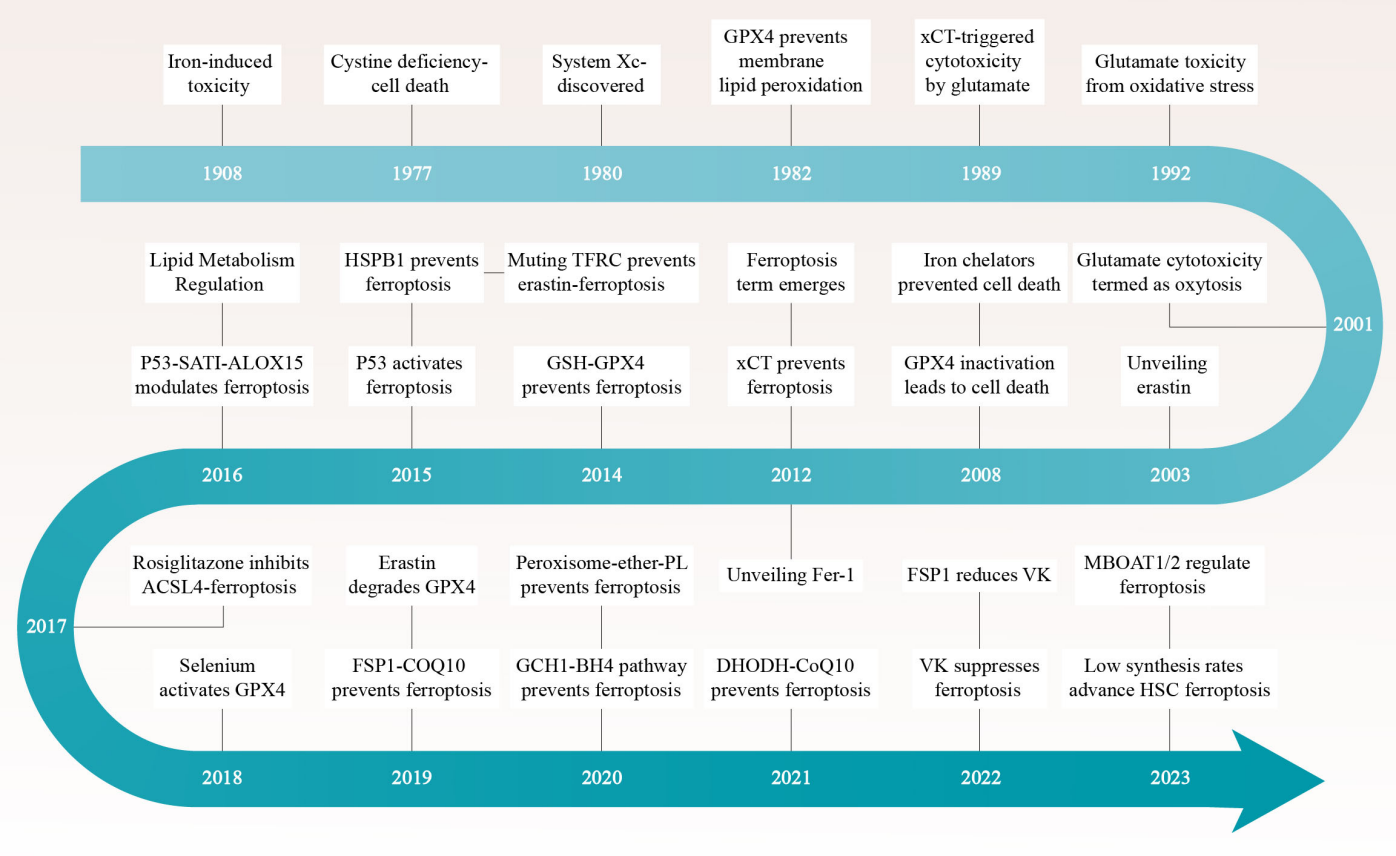
The ferric saga of ferroptosis unfolds through a compelling timeline dotted with pivotal breakthroughs. Each epoch is distinguished by seminal discoveries that have sculpted the narrative of our apprehension regarding this distinctive pathway of cellular demise. ACSL4, Acyl-CoA Synthetase Long-Chain Family Member 4; CoQ10, Ubiquinone-10; DHODH, Dihydroorotate Dehydrogenase; FSP1, Ferroptosis Suppressor Protein 1; GPX4, Glutathione Peroxidase 4; GSH, Glutathione; HSC, Hepatic Stellate Cells; MBOAT1/2, Membrane Bound O-Acylated Transferase Domain Containing 1/2; TFRC, Transferrin Receptor; VK, Vitamin K; xCT, Solute Carrier Family 7 Member 11.

In a groundbreaking study conducted in 2012, Dixon and colleagues unveiled a remarkable pathway of cell death that is intricately linked to the metabolism of iron, particularly within cancer cells (**Figure 1**) (3). They coined the term “ferroptosis” to describe this unique process of cellular demise, emphasizing its significance as it extends beyond mammals and is prevalent across various biological kingdoms, including plants, protists, and fungi (4, 5).

In specific terms, researchers confirmed the presence of ferroptosis in *Magnaporthe oryzae*, the rice blast fungus, through characteristics such as the accumulation of lipid peroxides detected by optimized fluorescence imaging technology and dependence on iron ions, which is crucial for the developmental cell death of conidia during appressorium maturation. Ferroptosis initially occurs in the terminal cell and subsequently affects the entire conidium. Furthermore, the article indicates that specifically inducing ferroptosis in rice can restrict the invasive growth of *Magnaporthe oryzae*, thus enhancing resistance to the rice blast disease (4).

Another study discovered that with increasing age, the levels of ferrous iron (Fe^2+^) rise and glutathione (GSH) levels decline in *Caenorhabditis elegans*, and this imbalance promotes the occurrence of ferroptosis. Treatment with ferroptosis inhibitors, such as Liproxstatin-1, can alleviate cell death caused by GSH depletion, indicating that ferroptosis plays a crucial role in the natural aging process of *Caenorhabditis elegans* (5).

This discovery not only enhances our understanding of cell death mechanisms but also offers a fresh perspective on how iron metabolism can influence the fate of cells in diverse organisms.

Ferroptosis is characterized by an overabundance of iron-dependent reactive oxygen species (ROS), a condition that is intimately associated with the diminished antioxidant defense provided by glutathione peroxidase 4 (GPX4) (3). When the cell’s antioxidant defenses are unable to counteract the overwhelming presence of ROS, ferroptosis ensues within the cell (6).

In this process, the peroxidation reaction not only triggers ferroptosis but also contributes to its further progression. Hence, the molecules and signaling pathways involved in iron metabolism and peroxidation reactions assume a pivotal role in the regulation of ferroptosis. The intricate interplay of these molecules and pathways significantly influences the maintenance of the cell’s antioxidant equilibrium and serves as a deterrent against the occurrence of ferroptosis.

In this review, we delve into the biological principles of ferroptosis and explore its application in various dermatological conditions. Specifically, we examine its specific impact on ichthyosis, psoriasis, polymorphous light eruption (PMLE), vitiligo, and melanoma. The review also encompasses targeted therapeutic strategies for the ferroptosis pathway and provides an evaluation of the potential and latest research trends surrounding these treatment methods in dermatological disease therapy.

## 2 Characteristics of ferroptosis

Iron ions are vital trace elements in the human body and are crucial for maintaining the normal functioning of living organisms (7). They play an irreplaceable role in the metabolic process of the skin, interacting with molecules such as ferritin within melanocytes to regulate cellular iron metabolism and redox balance (8). This interaction could have a profound impact on the function and viability of melanocytes.

However, excessive accumulation of iron ions can have detrimental effects. Through the Fenton reaction, excess iron ions can generate ROS, which can result in lipid peroxidation and serve as the pathological foundation for ferroptosis (9). Ferroptosis is a condition linked to iron metabolism dysfunction, and its development is intricately connected to the buildup of ROS and the state of iron overload.

**Figure 2:**
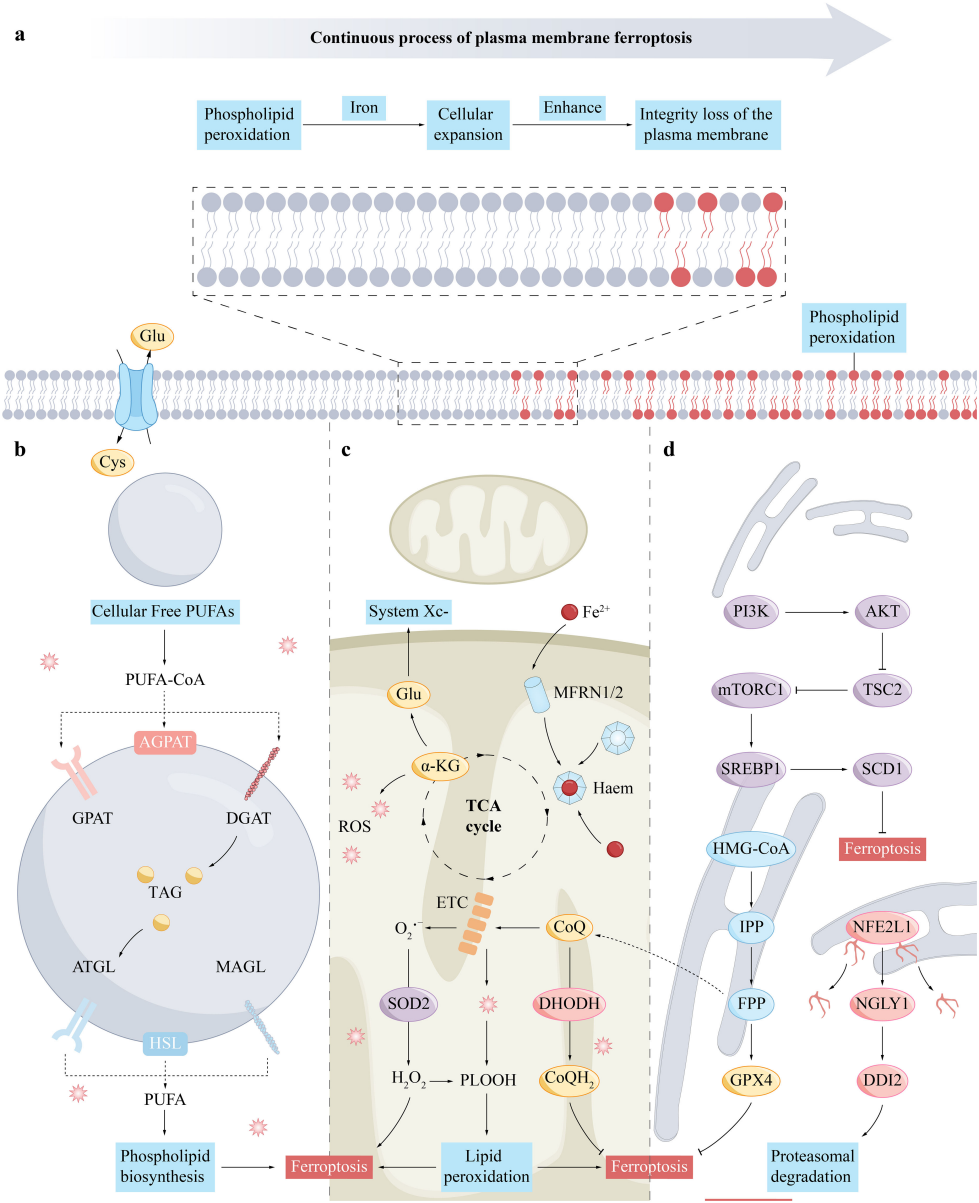
The mechanism of ferroptosis in the cell membrane **(A)**, lipid droplets **(B)**, mitochondria **(C)**, and endoplasmic reticulum **(D)**. **(A)**, Cell membrane rupture is a key event leading to cell death in the process of ferroptosis. It involves the accumulation of phospholipid hydroperoxides and the accumulation of iron ions, which can be produced through enzymatic (for example, via lipoxygenases) and non-enzymatic radical-mediated reactions. The susceptibility to ferroptosis is determined by the cell’s tendency to accumulate high levels of membrane lipid peroxides. **(B)**, Free PUFAs in the cytoplasm are converted into TAG through a process of fatty acid activation and are stored in lipid droplets. PUFAs esterified within TAG can be released from lipid droplets through lipolysis or lipophagy, with lipolysis involving a series of lipid droplet-associated lipases. **(C)**, The TCA cycle supplies electrons to the mitochondrial ETC, which partially reduces oxygen to ROS, particularly superoxide at complexes I and III. Superoxide can form hydrogen peroxide through dismutation and then produce hydroxyl radicals with Fe^2+^ in the Fenton reaction. Glutamine breakdown leads to α-ketoglutarate, which may enhance mitochondrial ROS and ferroptosis. MFRN1 and MFRN2 facilitate iron transport within the mitochondria, crucial for ROS production. CoQ, an essential electron carrier in the ETC, is synthesized in mitochondria and reduced by DHODH to CoQH2, which can either transfer electrons to complex III or act as an antioxidant to mitigate lipid peroxidation and ferroptosis. **(D)**, FPP contributes to CoQ synthesis, while IPP boosts selenoprotein production, including GPX4. SREBP1 and NFE2L1, released through proteolysis, regulate genes involved in lipid metabolism and oxidative stress in the nucleus. The PI3K-mTORC1 pathway enhances ferroptosis resistance by upregulating SREBP1 and SCD1. NFE2L1 is processed by NGLY1 and DDI2 after leaving the endoplasmic reticulum. AKT, Protein Kinase B; ATGL, Adipose Triglyceride Lipase; CoQ, Coenzyme Q10; CoQH2, Ubiquinol; DDI2, DNA Damage-Inducible 1 Homolog 2; DGAT, Diacylglycerol O-acyltransferase; DHODH, Dihydroorotate Dehydrogenase; ETC, Electron Transport Chain; FPP, Farnesyl Pyrophosphate; Glu, Glutamine; GPX4, Glutathione Peroxidase 4; HMG-CoA, Hydroxymethylglutaryl-Coenzyme A; IPP, Isopentenyl Pyrophosphate; MAGL, Monoacylglycerol Lipase; MFRN1/2, Mitoferrin 1 and 2; mTORC1, Mechanistic Target of Rapamycin Complex 1; NFE2L1, Nuclear Factor (Erythroid-derived 2)-like 1; NGLY1, N-glycanase 1; PI3K, Phosphatidylinositol-3 kinase; PLOOH, Phospholipid hydroperoxide; ROS, Reactive Oxygen Species; SCD1, Stearoyl-CoA Desaturase 1; SREBP1, Sterol Regulatory Element-Binding Protein 1; SOD2, Superoxide Dismutase 2.

In the regulatory mechanism of ferroptosis, numerous signaling pathways and molecules play crucial roles (**Figure 2**). These encompass the maintenance of iron homeostasis, the metabolic process of cysteine, the activity of GPX4, the synthesis of polyunsaturated fatty acids (PUFA), the regulatory role of nuclear factor E2-related factor (NRF2), the function of the tumor suppressor protein p53, the protective mechanism of heat shock proteins (HSPs), the regulatory role of iron regulatory inhibitor-1 (FSP1), the activation state of AMP-activated protein kinase (AMPK), and the supply of nicotinamide adenine dinucleotide phosphate (NADPH). These factors collectively form a complex regulatory network that influences the equilibrium of iron ions and the cellular health status.

### 2.1 Iron homeostasis

Iron is crucial for preventing lipid peroxidation in the process of ferroptosis (10). The mechanisms of iron uptake, intracellular transport, and storage are significant in regulating ferroptosis (11). Organisms carefully maintain iron homeostasis to ensure proper functioning. Transferrin receptor 1 (TFR1) plays a key role in absorbing iron ions and facilitating their conversion into ferrous ions within the cell, which is a critical step in preventing ferroptosis. Divalent metal transporter 1 (DMT1) releases ferrous ions into the intracellular iron pool, while ferritin light chain (FTL) and ferritin heavy chain 1 (FTH1) are responsible for safely storing excess iron ions in the cytoplasm, preventing their excessive accumulation (12). The proper expression and function of proteins related to iron metabolism such as hepcidin and ferroportin 1 (FPN1), are essential for controlling intracellular iron levels, as any imbalance can lead to increased iron levels, triggering metabolic issues (13, 14).

The skin serves multiple functions, acting as a protective barrier against external physical, chemical, and biological factors, while also playing roles in temperature regulation, sensory transmission, and immune responses (15). Iron homeostasis is particularly crucial for maintaining skin health, as iron ions are involved in various metabolic processes within skin cells (16). These processes include cell division, collagen synthesis, and antioxidant reactions. Optimal levels of iron ions help maintain the integrity of the skin barrier, facilitate wound healing, and support the regeneration of skin cells (17).

However, an imbalance of iron ions, whether in excess or deficiency, can have adverse effects on the skin microenvironment. Excessive iron ions can lead to oxidative stress, causing damage and triggering inflammatory reactions in skin cells (18). This, in turn, may accelerate the skin’s aging process and increase the risk of skin cancer. On the other hand, a deficiency of iron ions can impact the energy metabolism and function of skin cells, potentially resulting in dryness, fragility, and abnormal pigmentation of the skin (19).

### 2.2 Cysteine

Cystine/glutamate antiporter (System Xc-) is recognized as a critical cystine/glutamate antiporter within cellular systems. It is composed of two integral subunits, solute carrier family 7 member 11 (SLC7A11) and solute carrier family 3 member 2 (SLC3A2), which are chemically linked together by a disulfide bridge (20). This transport system crucially defends cells by absorbing cystine and releasing equal glutamate (21).

The proper functioning of this transporter is vital for cellular health. Initially, System Xc- facilitates cystine uptake through the interaction of its subunits SLC7A11 and SLC3A2 (22). Subsequently, within the cell, cystine is converted into cysteine, a crucial precursor for GSH synthesis (23). As a potent antioxidant, GSH, catalyzed by GPX4, transforms lipid peroxides into harmless compounds, safeguarding cells from oxidative damage.

In the skin microenvironment, the level of cysteine is strictly regulated. It is taken up from outside the cell through the System Xc- and then converted into cysteine for cellular use (24). The availability of cysteine affects the levels of GSH within skin cells, thereby influencing the skin’s resistance to environmental stress.

Cysteine’s anti-inflammatory properties in the skin are mediated through its role in the synthesis of glutathione, a potent antioxidant. This tripeptide helps neutralize ROS, which are often elevated during inflammatory responses and can damage skin cells. By reducing oxidative stress, cysteine indirectly suppresses the activation of the NF-κB pathway, a key transcription factor that drives the expression of pro-inflammatory cytokines such as TNF-α and IL-6 (25). The inhibition of NF-κB signaling by cysteine thus leads to a decrease in the production of these cytokines, which are central to the inflammatory process, ultimately contributing to the alleviation of skin inflammation.

### 2.3 GPX4

GPX4, a key enzyme in the glutathione peroxidase family, is essential for managing ferroptosis by catalyzing the reduction of lipid hydroperoxides and the conversion of GSH to glutathione disulfide (GSSG), thus preventing the lipid peroxidation associated with this form of cell death (26). It neutralizes detrimental lipid hydroperoxides into benign lipid alcohols, which is vital for preserving cell membrane integrity and shielding against oxidative stress (27).

Drugs like RAS Selective Lethal 3 (RSL3) specifically target GPX4, binding covalently to inactivate its function, leading to a buildup of lipid hydroperoxides that can trigger ferroptosis (28). The level of GPX4 expression dictates the cell’s vulnerability to ferroptosis; a decrease in its expression raises the risk, whereas an increase can bolster the cell’s defenses against ferroptosis (29).

GPX4 serves as a protective agent in the skin environment, contributing to skin health and vitality through its antioxidant properties. As a pivotal component of the skin’s antioxidant network, GPX4 can neutralize free radicals (30). These harmful agents are produced by everyday environmental factors such as ultraviolet radiation, pollutants, and stress. Furthermore, the activity of GPX4 is linked to the skin cells’ capacity to repair and regenerate, thus aiding in the restoration of the skin’s normal structure and function following damage (31).

### 2.4 PUFA

In the biological process of ferroptosis, PUFA are vital (32). They maintain cell membrane flexibility and fluidity, but their reactive double bonds make them susceptible to lipid peroxidation (33). The enzymes acyl-CoA synthetase long-chain family member 4 (ACSL4) and lysophosphatidylcholine acyltransferase 3 (LPCAT3), involved in PUFA synthesis, are critical, with their activity levels determining the cell’s susceptibility to ferroptosis.

Additionally, lipoxygenase (LOX), by promoting PUFA peroxidation, exacerbates ferroptosis (33, 34). Studies have found that decreasing LOX activity or expression levels can slow ferroptosis. Also, the concentration and distribution of free PUFA within the cell significantly influence the lipid peroxidation rate and ferroptosis severity (34).

PUFA are essential for skin health and functionality. They mitigate inflammation, crucial for managing skin conditions like eczema and psoriasis (35, 36). Moreover, they regulate skin cell growth and differentiation, promoting wound healing and skin regeneration (37). Since PUFA are oxidation-prone, their skin presence relates closely to the antioxidant defense mechanism (38–41). Skin’s antioxidant enzymes and molecules, like glutathione peroxidase (GPX) and vitamin E, shield PUFA from free radicals, ensuring skin health.

### 2.5 NRF2

In the mechanism of ferroptosis, NRF2 plays a central regulatory role (42). It acts as a key transcription factor within the cell to respond to oxidative stress, maintaining cellular iron metabolism and antioxidant status through a series of complex regulatory pathways (43).

Under conditions of oxidative stress, NRF2 can dissociate from the cytosolic protein Kelch-like ECH-associated protein 1 (KEAP) to which it is bound, and rapidly translocate to the nucleus (44). This process triggers the expression of a series of antioxidant and detoxifying enzyme genes, including members of the glutathione S-transferase family (GSTs), heme oxygenase 1 (HO-1), and quinone oxidoreductase 1 (NQO1) (45). The activation of these enzymes is crucial for neutralizing ROS within the cell and mitigating the damage caused by oxidative stress (46).

Moreover, NRF2 finely regulates key genes in the iron metabolism pathway, such as ferroportin, a protein that promotes the export of iron ions out of the cell (47). This action reduces the intracellular iron concentration and the generation of ROS caused by iron overload, thus lowering the risk of ferroptosis (48). Additionally, NRF2 also participates in the regulation of iron-binding proteins, such as the TFR1, which helps the cell more effectively manage the uptake and storage of iron (49).

NRF2 plays a pivotal role in maintaining skin health. As time progresses, the skin naturally undergoes an aging process, leading to a gradual decrease in NRF2 activity (50). This reduction in NRF2 expression levels diminishes the skin’s ability to defend against free radicals, thereby accelerating the external manifestations of skin aging, including wrinkles, sagging, and pigmentation (51). Consequently, this age-related decline in NRF2 activity significantly influences the skin aging process and merits attention (52).

NRF2 plays a multifaceted role in wound healing, inflammation management, and disease progression. In wound healing, NRF2 activation enhances the expression of antioxidant enzymes, which protect new cells from oxidative stress and facilitate the healing process (53). Additionally, NRF2 helps to regulate inflammation by inhibiting the production of inflammatory cytokines, thus reducing inflammation’s negative impact on wound recovery (54).

In the realm of inflammatory skin diseases, the role of NRF2 is pivotal, particularly in conditions like atopic dermatitis (AD) and psoriasis. In AD, the skin’s natural barrier function is compromised due to reduced NRF2 activity, which is crucial for maintaining the integrity of the skin. This weakening allows environmental irritants and allergens to penetrate more easily, leading to a heightened inflammatory response within the skin. The inflammation can manifest as redness, itching, and dryness, which are characteristic symptoms of AD (55). The lack of NRF2’s protective influence results in a prolonged and often distressing experience for those affected, as the skin’s ability to repair and regenerate is hindered.

In psoriasis, the situation is somewhat different but equally concerning. Here, NRF2’s diminished activity is implicated not just in the exacerbation of inflammation but also in the chronicity of the disease. The reduced NRF2 levels lead to an imbalance in the skin’s normal cell turnover process, causing skin cells to proliferate at an abnormally rapid rate. This over proliferation results in the formation of thick, scaly plaques that are a hallmark of Psoriasis. Moreover, the continuous cycle of rapid cell division and shedding contributes to the chronic nature of the condition, making it a long-term management challenge for patients (56). The cellular damage that accompanies this process can further disrupt the skin’s ability to function properly, leading to a range of symptoms that can significantly impact a person’s quality of life.

The connection between NRF2 and these inflammatory skin diseases underscores the potential for therapeutic interventions that target NRF2 activity. By enhancing NRF2’s function, it may be possible to strengthen the skin barrier in AD, reducing the susceptibility to irritants and allergens, and thereby mitigating inflammation. In Psoriasis, strategies to boost NRF2 activity could help normalize skin cell turnover, reducing the formation of plaques and the associated discomfort. Such interventions could offer a more effective approach to managing these conditions, improving the prognosis and overall well-being for individuals living with AD and Psoriasis.

Furthermore, NRF2’s dysregulation is implicated in the progression of certain skin cancers. In the early stages, abnormal NRF2 expression can lead to increased tumor cell proliferation and invasiveness, highlighting the importance of NRF2 in cancer development (57).

Understanding NRF2’s complex role in skin health is essential for developing targeted treatments. By modulating NRF2 activity, it may be possible to enhance wound healing, alleviate inflammation in conditions like AD and psoriasis, and potentially slow the progression of skin cancers. This underscores the need for further research into NRF2’s mechanisms and its potential as a therapeutic target in dermatology.

### 2.6 P53

The p53 protein serves a critical role in the cellular response to iron overload, maintaining a balanced state of iron by finely regulating the transcriptional activity of specific genes (58). Notably, p53 triggers the transcription of the ferroportin gene, a key membrane-bound protein that facilitates the transport of iron ions from inside the cell to the outside (59). This action effectively reduces cellular iron levels and decreases the production of ROS associated with iron overload. Additionally, p53 inhibits the expression of TFR1, thereby limiting cellular iron uptake and preventing the occurrence of iron overload (60).

Moreover, p53 is not only involved in iron homeostasis regulation but also enhances the cellular antioxidant defense system by activating the expression of a series of antioxidant enzymes, including GSTs, HO-1, and superoxide dismutase (SOD) (61). The activation of these antioxidant enzymes assists in neutralizing ROS, thereby protecting cells from oxidative stress damage.

When the skin is exposed to harmful environmental factors, such as ultraviolet rays, the p53 protein promptly activates its protective mechanism (62). It intervenes in the regulation of the cell cycle, triggering a pause in the cell cycle and providing valuable time for the cell to repair DNA damage (63). This timely cell cycle arrest not only prevents the proliferation of damaged cells but also effectively reduces the accumulation of genetic mutations, thereby significantly decreasing the incidence of skin cancer (64). Additionally, p53 promotes the synthesis of specific DNA repair enzymes, accelerating the repair of damaged DNA and maintaining the genetic stability of skin cells (65).

Moreover, p53 plays a pivotal role in combating skin aging. Over time, the activity of p53 within skin cells may gradually weaken, impacting the skin’s defense against external damage and decreasing the effectiveness of the antioxidant system (66). The decline in antioxidant capacity accelerates the external manifestations of skin aging, such as the loss of skin elasticity and the appearance of fine lines and wrinkles. Furthermore, the reduction in p53 activity may affect the renewal and repair capabilities of skin cells, further contributing to the aging process (67). Understanding the multifaceted role of p53 in skin health is crucial for developing strategies to maintain skin integrity and combat aging.

### 2.7 HSPs

In the context of ferroptosis, HSPs play a crucial role in regulating and protecting cells through diverse mechanisms. Specifically, heat shock protein family B (small) member 1 (HSPB1), a member of the HSP family, effectively mitigates the accumulation of iron ions within cells by limiting iron absorption. This not only alleviates oxidative stress damage to cells but also helps maintain the stability of the cytoskeleton (68). Additionally, heat shock protein family B (small) member 5 (HSPB5), another member of the HSP family, is notable for its location in the endoplasmic reticulum and significantly enhances the cell’s antioxidant capacity through its interaction with GPX4, providing an additional protective layer against the threat of ferroptosis (69).

The functions of HSPs extend beyond antioxidant and anti-inflammatory capacities: they also inhibit ferroptosis by safeguarding the health of mitochondria (70). Mitochondria, as the powerhouse of the cell, are vital for cell survival, and HSPs protect them from damage, thereby preventing the onset of ferroptosis (71). Furthermore, HSPs are involved in the regulation of various intracellular signaling pathways, including those closely related to cell survival and death, such as apoptosis and autophagy pathways (72). Maintaining the balance of these signaling pathways is crucial for the cell’s ability to resist ferroptosis.

HSPs are instrumental in combating skin aging and photoaging. Their presence enables skin cells to combat oxidative stress induced by environmental factors, particularly ultraviolet rays, thereby mitigating the skin damage that arises from prolonged ultraviolet (UV) exposure (73). The antioxidant capacity of HSPs plays a pivotal role in eliminating harmful free radicals, which have the potential to accelerate the aging process of the skin, resulting in a loss of elasticity and luster (74). Consequently, HSPs are indispensable for maintaining the health and youthful state of the skin, offering crucial support in preserving skin integrity.

Moreover, in the context of skin diseases such as psoriasis and contact dermatitis, the expression levels of HSPs may undergo substantial changes, influencing the severity of the disease and the effectiveness of treatment (75, 76). For instance, in psoriasis, an increase in the expression of HSPs may contribute to alleviating inflammatory responses, while in contact dermatitis, changes in HSP expression may be associated with the skin’s sensitivity to irritants. This underscores the significant role of HSPs in regulating the progression of skin diseases and influencing the response to treatment, thereby emphasizing their potential as targets for therapeutic interventions. Understanding the dynamic role of HSPs in skin health and disease progression is essential for developing tailored strategies to manage and treat various dermatological conditions.

### 2.8 FSP1

FSP1 plays a complex and multifaceted role in the process of ferroptosis, with its actions extending beyond the traditional GSH-GPX4 antioxidant pathway (77). What sets FSP1 apart is its unique ability to mitigate the effects of ferroptosis through a series of non-traditional pathways. For instance, it can influence the balance of iron ions within cells, regulate the storage and distribution of iron, and reduce the excessive accumulation of iron ions, thereby decreasing the incidence of ferroptosis (78).

An additional crucial function of FSP1 lies in its involvement in the NADH-dependent ubiquinone-oxidoreductase reaction, wherein ubiquinone is converted to ubiquinol, effectively inhibiting lipid peroxidation, a fundamental aspect of the ferroptosis process. As part of its function, ubiquinol has the capacity to neutralize free radicals, thereby interrupting the self-oxidation chain reaction of lipids (79). Simultaneously, it promotes the regeneration of the α-tocopherol radical, indirectly inhibiting lipid auto-oxidation and further slowing down the progression of ferroptosis (80).

FSP1, a multifunctional protein, serves as a protective shield for the skin against environmental stressors, including ultraviolet rays and pollutants, owing to its antioxidant and anti-inflammatory properties (81). Its pivotal role in balancing skin iron metabolism is crucial for preventing cell damage caused by iron overload, and it may also contribute to slowing down the skin’s aging process by supporting cell repair and regeneration (82). Moreover, the anti-inflammatory effects of FSP1 may aid in alleviating skin inflammatory conditions, while its involvement in ferroptosis offers promising perspectives for the prevention of skin cancer (83).

Furthermore, FSP1 may actively participate in maintaining the skin’s barrier function, which is indispensable for preventing the invasion of pathogens and allergens. In the realm of skin disease treatment, the regulatory role of FSP1 could emerge as a potential target for addressing inflammatory skin conditions such as psoriasis and venous ulcers (84). These insights not only enhance our comprehension of the role of FSP1 in skin physiology and pathology but also lay a scientific foundation for the development of novel strategies for skin protection and treatment.

### 2.9 AMPK

AMPK plays a pivotal role in regulating ferroptosis as the primary sensor of cellular metabolic status and energy levels (85). It is activated under conditions of glucose scarcity due to the increased ratio of intracellular adenosine monophosphate (AMP) to adenosine triphosphate (ATP) (86). The activation of AMPK helps prevent the occurrence of ferroptosis, while its inhibition increases the risk of ferroptosis.

In-depth research has revealed that the activation of AMPK is regulated by its upstream kinase liver kinase B1 (LKB1), a significant tumor suppressor, particularly in malignant tumors such as lung cancer, where mutations in LKB1 are common (87). Non-small cell lung cancer cells with LKB1 mutations exhibit high sensitivity to ferroptosis, suggesting that ferroptosis inducers may hold therapeutic potential for such tumors (88).

When cells encounter ferroptosis-inducing factors, the activation of AMPK not only inhibits the activity of its downstream acetyl-CoA carboxylase 1 (ACC1) but also reduces the synthesis of fatty acids (89). This process helps decrease the accumulation of intracellular lipid peroxides and slows down the progression of ferroptosis. Further analysis of lipid metabolism has confirmed that by inhibiting the synthesis of PUFA controlled by ACC1, AMPK plays a central role in inhibiting ferroptosis (89).

During the process of skin ferroptosis, the role of AMPK is particularly important. It not only stimulates the production of collagen, which is a key component in maintaining the structure and elasticity of the skin, but also enhances the skin’s firmness and elasticity through its activating effects, effectively combating signs of cell death (90).

In addition, the activation of AMPK also has anti-inflammatory effects, which can alleviate discomfort symptoms caused by skin inflammation, such as redness and itching (91). This anti-inflammatory action is particularly beneficial in alleviating the symptoms of certain skin diseases.

After the skin is damaged, the repair process is crucial. AMPK plays the role of an accelerator in this process. By stimulating cellular energy metabolism and accelerating cell proliferation, it promotes the rapid repair and regeneration of damaged skin tissue, thereby speeding up the healing of wounds (92).

### 2.10 Guanosine triphosphate cyclohydrolase 1

GCH1, an indispensable enzyme in the folate biosynthesis pathway, is intricately linked to ferroptosis (93). Its protective role stems from the metabolites it produces, tetrahydrobiopterin (BH4) and dihydrobiopterin (BH2), which are essential for cellular redox balance and the normal function of nitric oxide synthase (NOS) (94).

Beyond its involvement in folate generation, GCH1 also participates in the signaling of nitric oxide (NO) by converting guanosine triphosphate (GTP) to guanosine diphosphate (GDP) and NO. NO, a molecule with diverse biological functions, profoundly influences various physiological processes within the cell, including the regulation of cell death pathways (95). The antioxidant effect of NO is evident in its ability to react with the superoxide anion to form peroxynitrite (ONOO-), a reaction that reduces levels of ROS within the cell, thereby alleviating oxidative stress (96). However, in the context of ferroptosis, the excessive accumulation of NO or its derivatives may interact with iron ions, leading to lipid peroxidation and subsequent cellular damage.

In-depth analysis has unveiled another protective mechanism of GCH1 within the cell. Cells with higher GCH1 activity exhibit characteristics indicative of containing two chains of PUFA, which demonstrate the ability to shield the phosphatidylcholine on the cell membrane from oxidative damage. This protection may be achieved by enhancing the stability of the cell membrane and limiting the spread of ROS, thereby aiding the cell in resisting oxidative stress.

Although the specific biochemical pathways and molecular interactions of this protective mechanism are not fully understood, it is evident that GCH1 plays a significant role in maintaining cellular redox balance and fortifying the cell against oxidative stress (97).

In the face of the threat of ferroptosis, the antioxidant effect of NO assumes critical importance for skin cells (98). NO’s reaction with superoxide radicals to form ONOO- plays a pivotal role in neutralizing ROS, reducing oxidative stress, and providing a protective shield for cells, enabling them to resist the damage caused by ferroptosis (99).

Simultaneously, the indispensable role of GCH1 in maintaining the health of skin cells warrants attention (100). Involved in the biosynthesis of folate, a vital nutrient for cell division and DNA synthesis, GCH1 plays a key role in the renewal and repair processes of skin cells. Confronted with the challenge of ferroptosis, GCH1 ensures a stable supply of folate, promoting the rapid recovery and regeneration of damaged skin cells, thereby combating the cell damage caused by an imbalance of iron ions (101).

These multifaceted functions of GCH1 emphasize its central role in the skin’s defense system. Not only does it directly participate in the battle against ferroptosis through the antioxidant mechanism of NO, but it also indirectly promotes the health and regenerative capacity of skin cells by supporting the synthesis of folate. Together, these actions underscore the crucial role of GCH1 in safeguarding the skin from the effects of ferroptosis.

## 3 Ferroptosis in conjunction with other cellular demise mechanisms

Ferroptosis is a unique form of regulated cell death that relies on iron and sets itself apart from other forms such as apoptosis, necrosis, and autophagy (102–104). This regulated cell demise process is characterized by the build-up of lipid peroxidation products and the collapse of cellular defenses against oxidative stress, both induced by iron accumulation (105, 106). The distinctive role of iron in ferroptosis underscores the importance of comprehending the involved molecular pathways, as it presents a novel perspective on cell death that can be therapeutically targeted.

In stark contrast to apoptosis, which is a meticulously regulated process requiring energy and involving a protease cascade, ferroptosis is marked by the loss of membrane integrity due to oxidative damage (107). It also contrasts with necrosis, typically associated with uncontrolled cell swelling and rupture in response to severe injury or infection (108, 109). Autophagy, another cellular process involving the degradation and recycling of cellular components, also differs from ferroptosis. Drawing a clear distinction between ferroptosis and these other pathways is essential for deepening our understanding of cell death in various pathological conditions and for the development of innovative therapeutic strategies.

### 3.1 Ferroptosis and apoptosis

Apoptosis, governed by genetic factors, is essential for maintaining tissue equilibrium and immune function. Ferroptosis and apoptosis are two key regulatory processes within cells, playing complex roles in the pathogenesis of skin diseases, especially in their interactive effects within the skin microenvironment (110). Studies indicate a possible interaction between ferroptosis and apoptosis, with cells potentially transitioning from the iron-dependent, lipid peroxidation-characterized ferroptosis to apoptosis (111, 112). This shift can escalate cell susceptibility to apoptosis, and the tumor suppressor p53 can trigger ferroptosis, influencing tumor cell death and lifespan in preclinical models (113).

The connection between ferroptosis and apoptosis in skin diseases is multifaceted. For instance, the same stimuli that induce apoptosis, such as UV radiation or certain chemotherapeutic agents, can also promote ferroptosis in skin cells (114). Moreover, the molecular pathways that regulate these two forms of cell death can intersect. For example, the tumor suppressor protein p53, which is well-known for its role in inducing apoptosis, can also activate ferroptosis under certain conditions (115).

In the context of skin cancer, the balance between ferroptosis and apoptosis can influence tumor progression and response to therapy (116). An increased understanding of how these cell death pathways interact could lead to novel therapeutic strategies that target their intersection, potentially enhancing the efficacy of treatments and providing new options for patients with skin diseases.

### 3.2 Ferroptosis and necrosis

Necrosis, typically an accidental cell death process, can mimic apoptotic signaling, and ferroptosis, which is driven by iron and lipid peroxidation, often occurs with necrosis under certain conditions (117). Studies have shown increased mRNA levels of markers for both ferroptosis and necrosis in cell death induced by heme chloride, suggesting an interplay between these death mechanisms (118). Research has indicated that deficiencies in ACSL4, a lipid metabolism enzyme associated with ferroptosis, can lead to increased Mixed Lineage Kinase Domain-like Protein (MLKL), a protein involved in necrosis execution, making cells more susceptible to ferroptosis. This suggests a dynamic interrelation between the two pathways (119, 120). Additionally, NADPH, vital for cellular redox balance, is thought to influence both ferroptosis and necrosis, with its consumption during necrosis potentially sensitizing neighboring cells to ferroptosis due to reduced intracellular NADPH levels (121).

In the skin microenvironment, the relationship between ferroptosis and necrosis is particularly complex and can significantly influence the pathogenesis of skin diseases. Ferroptosis, characterized by iron accumulation and lipid peroxidation, can be triggered in the skin by various stimuli, including UV radiation and oxidative stress commonly encountered in dermatological conditions (122). Necrosis, often a result of acute cellular injury, can also occur in the skin following trauma or ischemia, leading to inflammation and tissue damage (123).

The interaction between these two modes of cell death can be bidirectional. For instance, the oxidative environment that promotes ferroptosis can also damage the cell membrane, potentially leading to necrosis (124). Conversely, the inflammatory response following necrosis can create a microenvironment that sensitizes cells to ferroptotic stimuli (125). This interplay may be particularly relevant in conditions like psoriasis, where both oxidative stress and cellular injury contribute to the disease pathology.

Moreover, the skin’s immune cells, such as macrophages and neutrophils, can release ROS and pro-inflammatory cytokines that can drive both ferroptosis and necrosis (116). The resulting cellular damage and death can further exacerbate skin inflammation and impair the skin’s barrier function, as seen in conditions like toxic epidermal necrolysis and bullous pemphigoid.

### 3.3 Ferroptosis and autophagy

Autophagy, involving lysosomal degradation of cellular components, plays a role in various diseases (126–128). It can trigger ferroptosis by degrading ferritin, leading to increased intracellular iron. This was observed in both fibroblasts and cancer cells, where autophagy enhanced ferroptosis through ferritin breakdown (129). Inhibition of autophagy-related genes like Autophagy-related protein 5 (Atg5) and Autophagy-related gene 7 (Atg7) reduced erastin-induced ferroptosis by lowering iron levels and lipid peroxidation (130, 131).

In inflammatory skin diseases such as psoriasis, the regulatory role of autophagy is particularly important, as it is responsible for clearing damaged organelles and proteins, helping to maintain cellular health and tissue homeostasis (132). Dysregulation of autophagy is associated with the abnormal cell proliferation and differentiation, as well as the exacerbation of inflammatory responses observed in psoriasis (133). Studies have found that abnormal autophagic activity in keratinocytes within the skin lesions of psoriasis may be related to the progression of the disease.

Within the skin microenvironment, the crosstalk between ferroptosis and autophagy may impact the inflammatory state and tissue repair of the disease. For instance, the activation of autophagy can alleviate inflammation, while the activation of ferroptosis may lead to the release of inflammatory mediators, intensifying the inflammatory response (134). Therefore, understanding how these two cellular processes interact is crucial for developing new strategies to treat skin diseases.

## 4 Ferroptosis and skin diseases

Ferroptosis, as a cell death mechanism triggered by the accumulation of intracellular iron ions and lipid peroxidation, is gradually revealing its importance in dermatological research. This mode of cell death is closely related to the pathogenesis of various skin pathological conditions, including but not limited to inflammatory skin diseases, autoimmune skin disorders, and skin tumors. In-depth research on ferroptosis has revealed its key role in the development of skin pathology, providing a new perspective for the diagnosis and treatment of skin diseases.

In the field of dermatopathology, the regulatory mechanisms of ferroptosis include the control of iron homeostasis, the production of ROS, and the balance of lipid metabolism. Disruptions in these mechanisms may lead to skin cell dysfunction or even death. For example, oxidative stress common in inflammatory skin diseases may exacerbate the accumulation of iron ions and lipid peroxidation, thereby triggering ferroptosis. In autoimmune skin diseases, the abnormal activation of the immune system may lead to iron metabolism imbalance, thus promoting the occurrence of ferroptosis. In skin tumors, ferroptosis may be related to the abnormal metabolism of tumor cells and changes in the microenvironment.

The multifaceted role of ferroptosis in dermatopathology emphasizes the necessity of in-depth research into its regulatory mechanisms. This not only helps us to better understand the pathogenesis of skin diseases but also provides the possibility for developing new treatment strategies, with the aim of improving patients’ skin health and quality of life.

### 4.1 Ferroptosis and ichthyosis

Ichthyosis, also referred to as keratosis, is an autosomal dominant genetic disorder characterized by dry, rough, and scaly skin that resembles fish scales (135). This condition typically manifests in infancy or childhood and may become more pronounced with age. The skin lesions associated with ichthyosis usually appear on specific parts of the body, including the extensor surfaces of the limbs, the back, and the buttocks (136).

Mutations in the LOX gene family, specifically arachidonic acid 12-lipoxygenase (ALOX12B) and arachidonate lipoxygenase 3 (ALOXE3), are common causes of ichthyosis (137). In the function of the skin barrier, arachidonic acid 12-lipoxygenase (12R-LOX) is responsible for catalyzing the conversion of arachidonic acid into 12R-Hydroperoxyeicosatetraenoic acid (12R-HPETE), and epidermal-type lipoxygenase-3 (eLOX3) further transforms 12R-HPETE into biologically active compounds (138). These compounds are involved in regulating the differentiation and function of skin cells, thus maintaining the skin’s barrier function. When mutations occur in the ALOXE3 or ALOX12B genes, this transformation process is disrupted, leading to abnormalities in the skin barrier function and consequently causing skin diseases such as non-bullous congenital ichthyosis (NCIE) (139).

**Figure 3:**
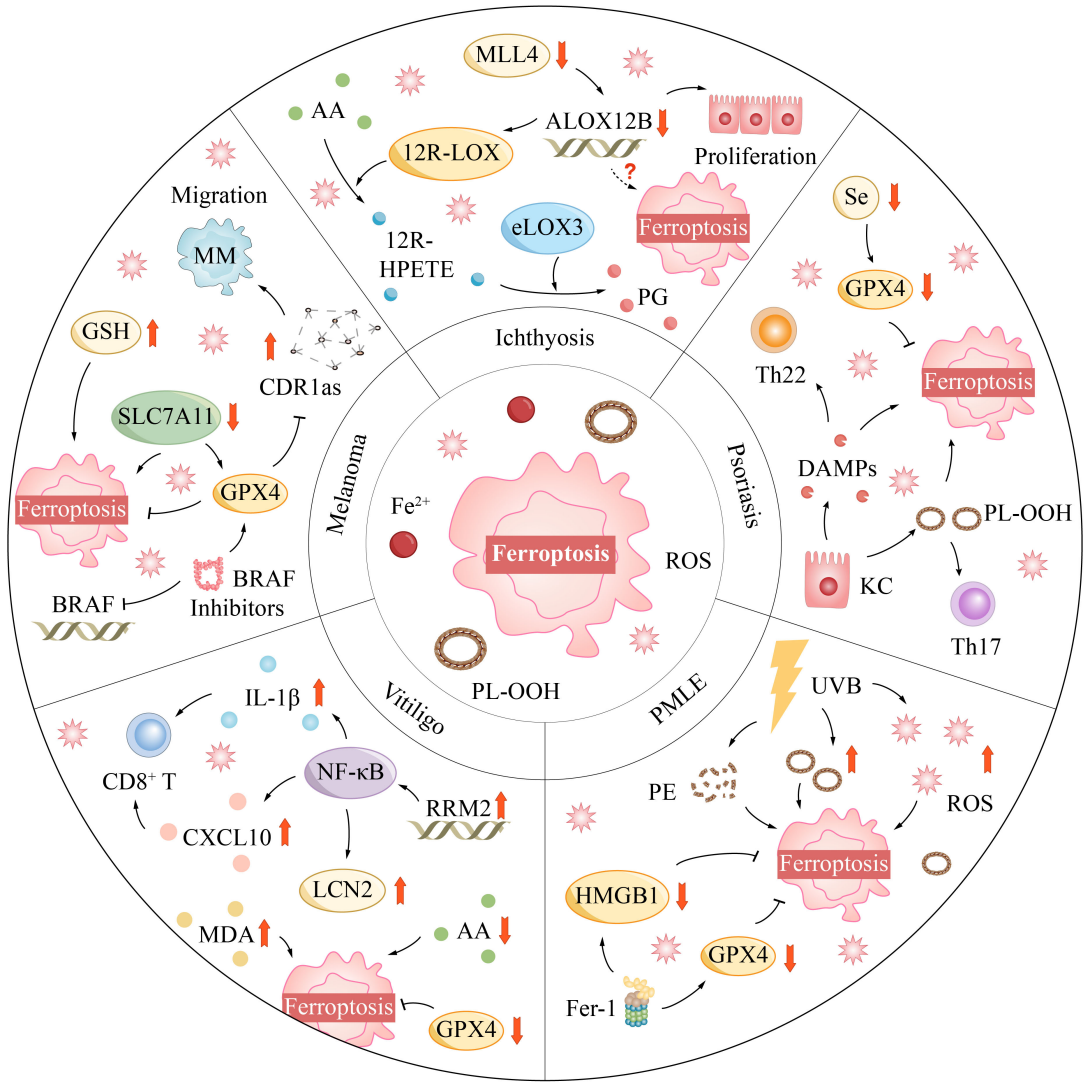
Ferroptosis mediates a variety of skin diseases.

Mixed Lineage Leukemia Protein-4 (MLL4), a pivotal histone methyltransferase, plays an essential role in biological processes such as cell differentiation and tumor suppression (140). Recent studies have revealed that MLL4 may regulate the process of ferroptosis by controlling the expression of the ALOX12B gene. In mouse models with specific knockouts of the MLL4 and ALOX12B genes, skin pathological changes similar to ichthyosis, including epidermal hyperplasia and desquamation, were observed (141). The expression of the ALOX12B gene was reduced in the epidermal tissues of these mice, suggesting a link to skin pathology (**Figure 3**).

Mice with functional loss mutations in the ALOX12B gene induced by ethylnitrosourea had red and shiny skin at birth, but quickly became dry and led to death, without exhibiting typical ichthyosis-like lesions (142). However, after the skin tissue of these mutant mice was transplanted into nude mice, local areas exhibited features similar to ichthyosis. Histological examination found that the epidermis of these skin grafts was significantly thickened, with abnormal keratinization and acanthosis, related to the pathological changes of ichthyosis (143). These research findings highlight the importance of MLL4 and ALOX12B in skin health.

### 4.2 Ferroptosis and psoriasis

Psoriasis is a chronic and recurring skin condition characterized by raised red patches covered with silvery scales, creating a clear boundary between affected areas and normal skin (144). It’s important to note that psoriasis is not contagious. Rather, it is caused by an overactive immune system leading to skin inflammation (145). Various factors, including emotional stress, infections, skin injuries, certain medications, and changes in body temperature, can trigger outbreaks or worsen psoriasis.

Ferroptosis, an iron-dependent form of regulated cell death, is closely connected to lipid peroxidation processes (146). This connection becomes especially pronounced in psoriasis patients, where skin lesions prominently display molecular markers of ferroptosis. Significantly, the keratinocytes within these lesions reveal abnormalities in lipid metabolism and an increased activation of lipid oxidation pathways, a stark contrast to healthy skin (147). These changes become even more evident at the single-cell level, where keratinocytes’ lipid oxidation activity displays a positive correlation with the Th22/Th17 pathway, demonstrating a temporal and dosage-sensitive dependency on ferroptosis (148).

In psoriasis, the interplay between ferroptosis and immune pathways may occur through various mechanisms. Ferroptosis is an iron-dependent form of cell death that involves the accumulation of lipid peroxidation, which is associated with inflammation and oxidative stress observed in psoriasis. Notably, ferroptosis may interact with the Th17/Th22 pathways in psoriasis, which play a crucial role in the immune response of the disease (149).

T helper 17 cells (Th17 cells) are key immune cells in the inflammatory response of psoriasis, promoting inflammation by producing Interleukin-17 (IL-17) and other cytokines (150). IL-17 can stimulate the proliferation and activation of keratinocytes, which is one of the pathological characteristics of psoriasis (151). Ferroptosis may regulate the immune response in psoriasis by affecting the function and survival of Th17 cells. For instance, inhibitors of ferroptosis, such as Ferrostatin-1, can reduce inflammatory responses in keratinocytes, which may be related to the modulation of Th17 cell functions (152).

Furthermore, in the pathogenesis of psoriasis, ferroptosis may impact the immune microenvironment by modulating the function of dendritic cells (DCs) and regulatory T cells (Tregs) (153). DCs are essential antigen-presenting cells in the immune system, playing a vital role in activating T cells and initiating immune responses (154). In psoriasis, DCs secrete cytokines like IL-23 and IL-12, promoting the differentiation and activation of Th17 cells (155). These Th17 cells then secrete IL-17 and IL-22, exacerbating inflammatory responses and the abnormal proliferation of keratinocytes (156).

The regulation of ferroptosis may affect the maturation and function of DCs. For example, the inhibition of ferroptosis may reduce the activation state of DCs, thereby diminishing their ability to activate Th17 cells (157). Additionally, ROS and lipid peroxidation products generated during ferroptosis may directly damage DCs, affecting their antigen presentation and immune regulatory functions.

Tregs are a subset of T cells with immune-suppressive functions. They suppress excessive immune responses and maintain immune tolerance by secreting anti-inflammatory cytokines such as IL-10 and transforming growth factor-beta (158). In psoriasis, the function of Treg cells may be suppressed, leading to an imbalance in immune regulation (159). Ferroptosis may further exacerbate this imbalance by affecting the stability and function of Treg cells. For instance, ferroptosis may induce apoptosis in Treg cells or alter their metabolic state, affecting their immune-suppressive functions (160).

Detailed investigations have highlighted alterations in expression levels of pivotal proteins like GPX4, ACSL4, Prostaglandin-endoperoxide synthase 2 (PTGS2), and Transferrin Receptor (TFRC) within psoriatic skin lesions. Of these, GPX4, an essential antioxidant enzyme, appears to have a reduced role in psoriasis pathogenesis (161). As selenium, a crucial element for GPX4 synthesis, is found deficient in psoriasis patients, this could potentially explain the decreased antioxidant capacity and increased susceptibility to ferroptosis in these individuals (162).

Furthermore, ferroptosis is not only implicated in direct cell destruction but also believed to trigger and amplify inflammatory responses by releasing damage-associated molecular patterns (DAMPs) (**Figure 3**) (163). This process activates immune cells and leads to an overproduction of inflammatory mediators, intensifying the chronic inflammatory environment within psoriatic lesions.

In summary, the relationship between ferroptosis and inflammation in psoriasis is complex. It involves abnormal lipid metabolism, changes in protein expression, and the escalation of inflammatory responses. Future research should strive to unravel the specific roles ferroptosis plays in psoriasis and explore the possibility of managing the disease’s inflammatory aspects by targeting the molecular mechanisms linked to ferroptosis.

### 4.3 Ferroptosis and PMLE

PMLE is a prevalent skin disease triggered by sun exposure. It is recognized by its distinctive skin lesions, which can present in varying forms such as erythema, papules, vesicles, or plaques, often accompanied by itching (164). The manifestations of PMLE typically appear on skin areas exposed to UV radiation, including the face, neck, upper limbs, and the backs of the hands (165).

UV radiation, notably in the ultraviolet radiation B (UVB) band, poses a significant threat to skin health. Research has demonstrated that UVB radiation can induce a process known as ferroptosis in keratinocytes, the surface cells of the skin (114). This process is characterized by an accumulation of ROS and lipid peroxides within cells under the influence of UVB radiation.

UVB radiation also triggers alterations in the lipid composition of keratinocytes, notably causing an accumulation of oxidized phosphatidylethanolamine (PE), a critical factor in the onset of ferroptosis. In this context, Ferrostatin-1 has shown its importance in cell models and human skin tissue samples exposed to UVB radiation. It can markedly reduce the release of high mobility group box-1 protein (HMGB1), further highlighting the crucial role of ferroptosis in cell necrosis and inflammatory responses (166).

Further investigations have revealed that both single and repeated UV radiation exposures can lead to lipid peroxidation accumulation and iron metabolism abnormalities in keratinocytes. Despite an increase in the expression of the antioxidant enzyme GPX4 and related antioxidant components after repeated radiation exposures, the cell’s defense against ferroptosis remains limited. This defense capability is notably weakened when there is an iron overload within the cells, suggesting that GPX4’s protective effect may be limited by the degree of oxidative damage.

Furthermore, nicotinamide mononucleotide, a precursor of NAD^+^, plays a vital role in regulating the redox imbalance caused by UVB radiation. Nicotinamide mononucleotide can stimulate the expression of GPX4, reducing the damage to mouse skin caused by UVB radiation (167). However, if GPX4 expression is inhibited, nicotinamide mononucleotide’s protective effect will be significantly affected, indicating that nicotinamide mononucleotide’s protective mechanism largely hinges on the normal function of GPX4 (**Figure 3**).

In conclusion, UV radiation significantly impacts skin health through various mechanisms, such as activating ferroptosis. These processes have implications not only on the survival of keratinocytes but also potentially influence the skin’s overall immune response by triggering the release of inflammatory mediators.

### 4.4 Ferroptosis and vitiligo

Vitiligo manifests as white patches appearing across various body parts, a distinct characteristic of this skin condition (168). It arises when melanocytes, the cells responsible for melanin production that lends color to the skin, cease to function or are destroyed. Consequently, this leads to a loss of skin color in the affected regions, resulting in conspicuous pale spots ranging in size from a few millimeters to several centimeters (169).

In clinical research, individuals with vitiligo have exhibited lower levels of arachidonic acid and reduced expression of the antioxidant enzyme GPX4 in their serum compared to healthy counterparts (170, 171). This decline in levels may heighten the susceptibility of melanocytes in the skin to ferroptosis and diminish the skin’s antioxidant capacity.

Further investigations have revealed that in vitiligo lesions, the upregulation of the ferroptosis-related gene RRM2 activates the NF-κB signaling pathway. This activation leads to an increased release of inflammatory factors such as CXCL10 and IL-1β, consequently recruiting CD8^+^ T immune cells to infiltrate the area and target melanocytes (172). Additionally, the activation of the NF-κB signal also results in the upregulation of LCN2, which not only accelerates the demise of melanocytes but also further amplifies the expression of RRM2.

Oxidative stress plays a pivotal role in the development of vitiligo, as it inflicts damage upon melanocytes through the accumulation of ROS, affecting the structural integrity and function of their DNA, lipids, and proteins (173). Skin lesions of vitiligo patients exhibit significantly elevated levels of hydrogen peroxide, reduced activity of antioxidant enzymes, and heightened levels of malondialdehyde, all serving as direct evidence of oxidative stress (174).

The impact of oxidative stress on melanocytes is multifaceted. It can induce the release of the protein HMGB1, disrupting the melanin synthesis process and leading to the downregulation of proteins related to melanin biosynthesis (**Figure 3**) (175). Furthermore, the excessive accumulation of ROS can disrupt the synthesis and stability of lipids within melanocytes, damage the mitochondrial electron transport chain, and initiate a detrimental cycle (176).

Moreover, melanocytes display a greater propensity for iron uptake than keratinocytes. Under external oxidative stress, the level of Fe^2+^ escalates, significantly heightening the sensitivity of melanocytes to ferroptosis (177). Specific triggers of ferroptosis, such as erastin, may promote this process by affecting the cystine/glutamate antiporter system or by inhibiting the activity of GSH peroxidase (178).

### 4.5 Ferroptosis and melanoma

Melanoma, a type of skin cancer, originates from melanocytes, the cells responsible for producing the pigment melanin that gives color to the skin (179). It stands out as one of the most aggressive forms of skin cancer and has the potential to metastasize to other parts of the body if not identified and treated promptly. Typically, melanoma is distinguished by the presence of a new or evolving mole or skin lesion, frequently exhibiting irregular borders, diverse colors, and occasionally an elevated surface (180).

Recent scientific investigations have brought to light the critical role of cerebellar degeneration-related protein 1 antisense (CDR1as) in the differentiation process of melanoma cells. Evidence has shown that the absence of CDR1as is directly associated with the heightened metastatic potential of melanoma cells, while cells expressing higher levels of CDR1as demonstrate increased sensitivity to GPX4 inhibitors (181). This revelation suggests that CDR1as could serve as a pivotal molecular target for inducing ferroptosis in melanoma cells (**Figure 3**).

Moreover, further research, including studies by prominent researchers like Sato, has indicated that the malfunction of System Xc- results in reduced cystine absorption by melanoma cells, leading to decreased GSH levels (182). Consequently, this hinders the cells’ ability to proliferate *in vitro*, migrate, and generate tumors and metastases *in vivo*. Although these experimental findings underscore the potential involvement of the lymphatic system in melanoma metastasis, our understanding of the specific mechanisms remains incomplete (183).

Additional experiments have observed heightened levels of GSH and a higher GSH/GS-SG ratio in the bloodstream of melanoma cells, suggesting a more oxidative blood environment that could potentially promote ferroptosis. The loss of GPX4 function diminishes tumor formation via intravenous injection, a phenomenon reversible by pretreatment with ferroptosis inhibitors. Notably, lymph node metastasis remains unaffected, indicating a potential direct protective effect of lymph fluid against ferroptosis (184).

Furthermore, the mutation status of the BRAF gene and the use of BRAF inhibitors both influence the oxidative metabolism and oxidative phosphorylation levels of melanoma cells, potentially determining their sensitivity to ferroptosis inducers (185). Specifically, the downregulation of SLC7A11 and increased reliance on glutamine metabolism signify heightened sensitivity to ferroptosis in metastatic melanoma cells (186). BRAF inhibitors such as vemurafenib, by modulating lipid metabolism and enhancing dependence on GPX4, offer novel insights into addressing drug resistance in targeted melanoma therapy (187).

In the realm of immunotherapy for melanoma, PD-1 inhibitors activate CD8^+^ T cells, inducing the secretion of IFN-γ (188). This process reduces the demand for cystine and GSH synthesis in melanoma cells, thereby augmenting the specific lipid peroxidation effect in ferroptosis. This discovery signifies that treatment strategies targeting the ferroptosis pathway can significantly enhance the efficacy of immunotherapy.

Concurrently, high expression levels of TYRO3 are associated with resistance to PD-1 inhibitors, and inhibiting TYRO3 can promote tumor cell ferroptosis (189). Additionally, reducing NRF2 levels can facilitate the infiltration of CD8^+^ and CD4^+^ T cells, and combining anti-PD-1 treatment can further bolster the therapeutic effect against melanoma (190).

## 5 Therapeutic approaches targeting ferroptosis in skin disorders

The identification of biomarkers is a critical step in the clinical diagnosis of diseases involving ferroptosis. These biomarkers can include specific lipid peroxidation products, altered iron levels, or changes in the expression of genes and proteins involved in ferroptosis. For instance, elevated levels of malondialdehyde (MDA) or 4-hydroxynonenal (4-HNE), which are lipid peroxidation markers, could indicate ongoing ferroptosis. Additionally, the assessment of iron regulatory proteins like ferritin and transferrin can provide insights into the status of iron homeostasis.

Diagnostic tools tailored to detect ferroptosis-related biomarkers are essential for clinical practice. Advanced imaging techniques, such as MRI with iron-sensitive contrast agents, can help visualize iron accumulation in tissues. Moreover, the development of assays to quantify lipid peroxidation products in blood or tissue samples can serve as a non-invasive diagnostic method. Liquid biopsies, which involve the analysis of circulating cell-free DNA, proteins, or extracellular vesicles, may also reveal signatures of ferroptosis.

In skin diseases, histopathological examination is a valuable diagnostic tool. The presence of ferroptotic cells in skin biopsies can be identified by characteristic morphological changes, such as the accumulation of lipid droplets, the presence of iron deposits, and the absence of typical apoptotic or necrotic features. Specialized staining techniques, like the use of Prussian blue for iron detection or the detection of specific lipid peroxidation products, can further aid in the diagnosis.

The therapeutic potential of ferroptosis presents vast opportunities in the treatment of skin diseases. Through the regulation of iron metabolism, inhibition of ROS generation, induction of ferroptosis, and integration of immunotherapy, it holds promise for providing patients with more effective and safer treatment options.

For example, the utilization of iron chelators, such as deferoxamine, can effectively diminish the concentration of intracellular iron ions, thereby reducing iron-dependent ROS production (191). Additionally, antioxidants like vitamin E and selenium can play a pivotal role in curtailing the accumulation of ROS, thereby safeguarding skin cells from oxidative damage (192).

Moreover, immunotherapy has demonstrated significant potential in the treatment of skin diseases. By activating immune cells in the skin, particularly CD8^+^ T cells, it can bolster immune surveillance and the clearance of tumor cells, thereby enhancing treatment efficacy (193).

### 5.1 Therapeutic approaches targeting ferroptosis in ichthyosis

Targeting ferroptosis for the treatment of ichthyosis presents an emerging therapeutic strategy. Utilizing iron chelators to reduce intracellular iron ion levels can decrease the production of hydroxyl radicals through the Fenton reaction, thereby inhibiting the accumulation of ROS (194). Additionally, modulating MLL4 and ALOX12B may prove to be an effective approach to treating ichthyosis. As a histone methyltransferase, MLL4 potentially influences the process of ferroptosis by regulating the expression of ALOX12B (141). The skin pathological changes observed in mouse models with knockouts of the MLL4 and ALOX12B genes, which resemble ichthyosis, provide experimental evidence supporting the treatment of ichthyosis through targeting the ferroptosis pathway.

To further expand on the relationship between ferroptosis and ichthyosis, it is important to consider the role of genetic factors in the development of the disease. Ichthyosis is a group of genetic disorders characterized by abnormal skin barrier function and abnormal desquamation, leading to the accumulation of scale-like skin. The identification of genes associated with ichthyosis, such as MLL4 and ALOX12B, has opened up new avenues for understanding the molecular mechanisms underlying the disease. MLL4, as a histone methyltransferase, is involved in the regulation of gene expression, including that of ALOX12B, which is implicated in skin barrier function and inflammation (195).

The modulation of these genes could potentially influence the susceptibility of skin cells to ferroptosis, thereby affecting the progression and severity of ichthyosis. This is supported by the observation of skin pathological changes in mouse models with knockouts of MLL4 and ALOX12B genes, which exhibit features similar to ichthyosis (196). These findings suggest that targeting the ferroptosis pathway may not only help in understanding the pathogenesis of ichthyosis but also in developing novel therapeutic strategies.

Moreover, the use of iron chelators and other compounds that can modulate the ferroptosis pathway could provide a novel therapeutic approach for ichthyosis. By reducing intracellular iron levels and consequently the production of ROS, it may be possible to ameliorate the symptoms of ichthyosis and improve skin health (197). This approach is in line with the current understanding of the role of oxidative stress in the pathogenesis of various skin diseases, including ichthyosis.

In conclusion, the targeting of ferroptosis in ichthyosis represents a promising area of research with potential therapeutic implications. Further studies are needed to fully understand the complex interplay between genetic factors, oxidative stress, and cell death pathways in the context of ichthyosis, which could lead to the development of more effective treatments for this group of disorders.

### 5.2 Therapeutic approaches targeting ferroptosis in psoriasis

Addressing psoriasis through the lens of ferroptosis presents a pioneering therapeutic strategy, concentrating on modulating cellular demise pathways intricately tied to iron homeostasis and ROS dynamics (198, 199). This strategy employs iron ion chelators to reduce the iron content within cells, thereby alleviating oxidative stress reactions and combating the pathological changes associated with psoriasis (200). Simultaneously, by targeting the activity of key enzymes such as GPX4, it can induce pathological skin cells to undergo ferroptosis, offering a novel therapeutic approach to control the progression of psoriasis (147).

To further expand on this strategy, it is important to consider the implications of ferroptosis in the clinical diagnosis and prognosis of psoriasis. The activation of ferroptosis in psoriatic lesions has been observed, with decreased expression of GPX4 and increased levels of lipid peroxidation markers, such as 4-HNE (199). This suggests that assessing the levels of these markers could potentially serve as diagnostic indicators of psoriasis. Moreover, the modulation of ferroptosis-related genes and pathways could provide a basis for developing prognostic biomarkers and therapeutic targets.

The use of iron chelators and other compounds that target the ferroptosis pathway, such as small molecule compounds predicted to target key genes, may offer new avenues for treatment (147). These compounds could be used to modulate the expression of genes associated with ferroptosis, potentially leading to improved disease management and outcomes for patients with psoriasis.

In conclusion, the integration of ferroptosis into the therapeutic strategy for psoriasis is a promising development. By targeting the molecular mechanisms underlying ferroptosis, it may be possible to develop more effective treatments and improve the prognosis for individuals with psoriasis. Further research is needed to fully understand the role of ferroptosis in psoriasis and to translate these findings into clinical practice.

### 5.3 Therapeutic approaches targeting ferroptosis in PMLE

UVB radiation plays a pivotal role in inducing ferroptosis in PMLE, leading to an accumulation of oxidized PE, which is closely associated with the onset of ferroptosis (166). Notably, Ferrostatin-1 demonstrates the ability to reduce the release of HMGB1 in UVB-irradiated cells, highlighting the significance of ferroptosis in cell necrosis and inflammation. Despite an increase in GPX4 expression, repeated UV radiation can still induce lipid peroxidation and disrupt iron metabolism, particularly under conditions of iron overload (167). Furthermore, nicotinamide mononucleotide, acting as a precursor of NAD^+^, regulates the redox imbalance caused by UVB and stimulates GPX4 expression, thereby reducing skin damage in UVB-exposed mice. However, the protective effect of nicotinamide mononucleotide is significantly compromised if GPX4 expression is inhibited, indicating that the protective mechanism of nicotinamide mononucleotide largely relies on the normal function of GPX4. A deeper understanding of the role of ferroptosis in skin pathology may pave the way for the development of more precise targeted therapies in the future, potentially enhancing treatment outcomes and the quality of life for PMLE patients (164, 201).

A deeper understanding of the role of ferroptosis in skin pathology may pave the way for the development of more precise targeted therapies in the future, potentially enhancing treatment outcomes and the quality of life for PMLE patients (202). The exploration of ferroptosis-related genes in the context of PMLE could provide valuable insights into the disease’s pathology and identify potential therapeutic targets. For instance, the differential expression of FRGs in response to UVB radiation could serve as a biomarker for disease activity and prognosis, aiding in the development of personalized treatment strategies (203).

In conclusion, the integration of ferroptosis into the clinical management of PMLE could lead to more personalized and effective treatment approaches. Further research is needed to fully elucidate the role of ferroptosis in PMLE and to translate these findings into clinical practice.

### 5.4 Therapeutic approaches targeting ferroptosis in vitiligo

Targeting ferroptosis in vitiligo treatment is an emerging strategy that focuses on the modulation of GPX4 levels to reduce melanocyte susceptibility to ferroptosis (204). In vitiligo lesions, the upregulation of the RRM2 gene activates the NF-κB signaling pathway, leading to increased inflammation and melanocyte damage. The accumulation of ROS due to oxidative stress not only harms melanocytes but also elevates oxidative stress markers in skin lesions. Melanocytes, with their high iron uptake capacity, are particularly vulnerable to oxidative stress, increasing the risk of ferroptosis. Compounds such as erastin may promote ferroptosis through distinct mechanisms, further impacting melanocyte health.

The study by Wu et al. (204) provides evidence that altered expression of ferroptosis markers and dysregulated iron metabolism may play a role in vitiligo pathogenesis. The findings suggest that erastin can induce ferroptosis in melanocytes, which can be mitigated by N-acetyl-L-cysteine (NAC) treatment, indicating a potential therapeutic approach to protect melanocytes from ferroptosis.

Moreover, the research by Zhang et al. (205) identified RNA-binding protein SLC3A2 as a regulator of melanocyte ferroptosis in vitiligo through integrated analysis of single-cell and bulk RNA-sequencing. This study revealed that downregulating SLC3A2 can promote ferroptosis in melanocytes, offering new insights into the pathogenesis of vitiligo and potential therapeutic targets.

In conclusion, understanding the role of ferroptosis in vitiligo and targeting key regulators such as GPX4 and SLC3A2 may lead to the development of novel treatments that could improve outcomes for patients with vitiligo.

### 5.5 Therapeutic approaches targeting ferroptosis in melanoma

Recent research advancements have significantly illuminated the pivotal role of ferroptosis in the pathogenesis and treatment strategies of melanoma (206, 207). With a comprehensive understanding of this process, scientists have developed a range of new drugs for the treatment of melanoma, primarily categorized as molecularly targeted therapies and immune checkpoint inhibitors. In the realm of molecular targeted therapy, BRAFV600E inhibitors hold particular significance. Given the prevalence of BRAF gene mutations in many melanoma patients, BRAFV600E inhibitors effectively curb the growth of tumor cells by specifically targeting the activity of this mutated gene (208). Additionally, immune checkpoint inhibitors, such as anti-programmed death protein 1 (PD-1) antibodies, have demonstrated noteworthy therapeutic effects (209). These drugs bolster the body’s natural defense against tumors by alleviating the suppression of the immune system by tumor cells.

Ferroptosis, an iron-dependent form of cell death, has emerged as a potential therapeutic target in melanoma. The role of ferroptosis in melanoma is multifaceted, influencing the disease’s progression, metastasis, and response to treatment. Studies have shown that certain melanoma cells are sensitive to ferroptosis inducers like erastin and RSL3, which could be exploited for therapeutic benefit (210). Furthermore, the sensitivity of melanoma cells to ferroptosis has been linked to their dedifferentiation status, with dedifferentiated cells being more susceptible to ferroptosis, suggesting a potential for combined treatment with BRAF inhibitors and ferroptosis inducers (211).

The manipulation of ferroptosis pathways could also enhance the efficacy of immunotherapies. For instance, the loss of GPX4, a key enzyme in ferroptosis, has been shown to promote an immune response against melanoma cells, indicating that ferroptosis may play a role in antitumor immunity (212). Additionally, the expression of certain microRNAs, such as miR-137 and miR-9, has been found to regulate ferroptosis in melanoma by targeting glutamate metabolism, which could have implications for therapy resistance and sensitivity.

In conclusion, the interplay between melanoma, ferroptosis, and the immune system is complex and holds promise for the development of novel therapeutic strategies. Targeting ferroptosis in melanoma may offer a new avenue for treatment, either as a standalone approach or in combination with existing therapies. As research in this field progresses, a better understanding of the mechanisms underlying ferroptosis and its role in melanoma will be crucial for the design of effective treatment strategies.

The advent of these new drugs marks substantial progress in the treatment of skin diseases. They have not only enhanced patients’ survival rates but also their quality of life. With ongoing research and clinical trials, the anticipation of developing more treatment options and strategies in the future brings renewed hope to patients.

## 6 Conclusion

Ferroptosis, as a distinct cell death mechanism, is gaining recognition for its potential role in skin disease pathology. Our review highlights the need for further exploration into how ferroptosis intersects with conditions such as psoriasis, vitiligo, and melanoma. While the therapeutic potential of targeting ferroptosis is promising, particularly with drugs that modulate iron metabolism and lipid peroxidation, it is clear that our understanding of these interactions is still evolving.

The development of targeted therapies, possibly enhanced through nanotechnology, could offer novel treatment options for skin diseases. However, the complexity of ferroptosis and its varied implications across different pathologies underscore the necessity for cautious optimism and continued research. This includes addressing current knowledge gaps, refining drug delivery systems, and assessing the long-term safety and efficacy of ferroptosis-targeting treatments.

Looking ahead, the integration of advanced technologies such as gene editing and immunomodulation may provide additional avenues for manipulating ferroptosis in a clinical setting. As our understanding of ferroptosis deepens, so too will the potential for innovative treatments that could significantly impact the lives of those affected by skin diseases.
